# Altered Serum Tumor Necrosis Factor and Interleukin-1β in First-Episode Drug-Naive and Chronic Schizophrenia

**DOI:** 10.3389/fnins.2018.00296

**Published:** 2018-05-11

**Authors:** Furong Zhu, Lulu Zhang, Fang Liu, Renrong Wu, Wenbin Guo, Jianjun Ou, Xiangyang Zhang, Jingping Zhao

**Affiliations:** ^1^Mental Health Institute of the Second Xiangya Hospital, Central South University, Chinese National Clinical Research Center on Mental Health Disorders, Chinese National Technology Institute on Mental Disorders, Hunan Key Laboratory of Psychiatry and Mental Health, Changsha, China; ^2^Department of Psychiatry, Guangzhou First People's Hospital, the Second Affiliated Hospital of South China University of Technology, Guangzhou, China; ^3^First Affiliated Hospital of Kunming Medical University, Kunming, China; ^4^Department of Psychiatry and Behavioral Sciences, UT Houston Medical School, The University of Texas Health Science Center, Houston, TX, United States; ^5^Guangzhou Hui Ai Hospital, Affliated Brain Hospital of Guangzhou Medical University, Guangzhou, China

**Keywords:** schizophrenia, cytokines, TNF-α, IL-1β, negative symptoms

## Abstract

**Objective:** Abnormality of the immune system might play a significant role in the pathogenesis of schizophrenia. We want to identity whether the serum TNF-α and IL-1β levels were changed in FEDN patients and CP and to investigate the relationship between both cytokines and psychopathological symptoms.

**Methods:** We recruited 69 FEDN patients, 87 CP and 61 healthy controls. Schizophrenia symptomatology was evaluated with the Positive and Negative Syndrome Scale (PANSS), the Scale for the Assessment of Negative Symptoms (SANS) and Clinical Global Impression Scale (CGI). Serum TNF-α and IL-1β levels were examined using sandwich enzyme-linked immunosorbent assay (ELISA).

**Results:** TNF-α and IL-1β levels in CP were significantly higher compared to healthy controls, but TNF-α and IL-1β levels in FEDN patients were significantly lower than in both CP and healthy controls. A moderate correlation between serum TNF-α or IL-1β levels and PANSS negative subscore was found in CP. But there was no correlation between altered cytokines and clinical symptoms in FEDN patients.

**Conclusions:** Increased TNF-α and IL-1β levels in chronic patients may be associated with the progression, psychotropic drugs or other factors occur during chronic stage. Immune modulating treatments may become a new strategy of therapy for this subgroup of patients.

## Introduction

Schizophrenia is a chronic and severe mental disorder with significant impairment in psychosocial functioning. The mechanisms of schizophrenia are essentially unclear. More and more evidence suggest that abnormal immune system and immunological responses may be related with the etiology of schizophrenia'; (DeLegge and Smoke, 2008; Miller et al., 2011; Monji et al., 2013). Treatment of anti-inflammatory medications for schizophrenia has further supported that neuroinflammation may contribute to the etiology of this disorder (Sommer et al., 2014; Goldsmith et al., 2016).

Cytokines are the important messengers between the central nervous system (CNS) and immune cells. They play an important role not only in the cell-cell communication but also in the function of the immune system in the central nervous system (CNS) (Müller et al., 2015). A number of cytokines such as tumor necrosis factor (TNF)-α, interleukin (IL)-2, IL-1, and IL-6 have been found to involve in neuro-immune-endocrine communication and regulate neuronal activities in the mature CNS (Behrens et al., 2008; Fan et al., 2015; Schiavone and Trabace, 2017; Schiavone et al., 2017). Previous data have found altered levels of cytokines in the cerebrospinal fluid and the peripheral blood of patients with schizophrenia (Garver et al., 2003; Potvin et al., 2008; Rodrigues-Amorim et al., 2017), suggesting that cytokines may play an essential role in the etiology of schizophrenia (Fan et al., 2007; Song et al., 2009).

TNF-α and IL-1β are the proinflammatory cytokines. Both TNF-α and IL-1β play essential roles in the immune response because they promote dopaminergic neuronal differentiation of neural stem cells and regulate the development of dopamine neurons (Rodriguez-Pallares et al., 2005). They are also participated in the selective vulnerability of the nigrostriatal pathway related with dopaminergic neurotoxicity (Ferrari et al., 2006; Sriram et al., 2006). Both TNF-α and IL-1β were among the mostly reported cytokines in schizophrenia. For example, Liu et. al reported that schizophrenia patients had significantly overexpressed TNF-α and IL-1β in blood mononuclear cells (Liu et al., 2010). Recently, one study found that TNF-α and IL-1β were increased in the blood of first onset and acute relapse patients with schizophrenia (Wang et al., 2014). We found significantly increased TNF-α and IL-1β levels in an immune-related animal model that imitated negative symptoms in schizophrenia in our recent study (Zhu et al., 2014a). It has been hypothesized that increased levels of TNF-α and IL-1β may elevate the immune responses of other cytokines, resulting in an imbalance of Th1/Th2 cytokines in schizophrenia (Müller et al., 2000).

But until now, no study has simultaneously reported TNF-α and IL-1β in both first-episode drug-naïve (FEDN) and chronic patients (CP) with schizophrenia. In this study we wanted to know whether the serum TNF-α and IL-1β levels were changed in FEDN patients and CP, and we also aimed to investigate the relationship between the both cytokines and psychopathological symptoms.

## Methods

### Subjects

Sixty-nine (male/female = 46/23) FEDN patients and 87 (male/female = 44/43) CP who met DSM-IV criteria for schizophrenia were recruited from the First Affiliated Hospital of Kunming Medical University and Guangzhou Baiyun Psychiatric Hospital. They included both inpatients and outpatients. The inclusion criteria for FEDN patients were: (1) between 18 and 45 years; (2) course of illness ≤ 2 years; (3) naïve to all psychotropic medications; (4) a stable living arrangement. The inclusion criteria for CP were: (1) aged 18–45 years; (2) course of illness ≥ 5 years; (3) on psychotropic medications; (4) able to understand the process of the study. The exclusion criteria were: (1) a psychiatric diagnosis other than schizophrenia (determined by SCID); (2) serious or unstable medical conditions including heart disease, epilepsy, hepatic or renal diseases, diabetes, aplastic anemia, systemic lupus erythematous or asthma; (3) planning to become pregnant, or were pregnant or breastfeeding. (4) Subjects with ongoing infections, allergies or past history of autoimmune disorders. (5) subjects suffered from substance abuse/dependence other than tobacco (which was based on subject and family report), received immunosuppressive drugs, or took medications for physical diseases.

Sixty-one healthy subjects (male/female = 31/30) were recruited from the local community in Kunming and Guangzhou. A clinical psychiatrist assessed the mental status and family history of any psychiatric disorder of the healthy controls. All of the healthy controls had no history of psychiatric diseases and a family history of psychiatric disorder. The other details of the exclusion criteria of healthy controls are the same as the patients' exclusion criteria except number one.

We obtained a complete medical history, physical examination and laboratorial tests from patients and control subjects. All subjects were Han Chinese and gave signed informed consent to participate in the study. The study protocol was approved by the ethics committee of the First Affiliated Hospital of Kunming Medical University and Guangzhou Baiyun Psychiatric Hospital. Then we gained the complete medical history, physical examination and laboratorial examination.

### Clinical measures

Two experienced psychiatrists evaluated patients' symptoms by PANSS and the Scale for the Assessment of Negative Symptoms (SANS). Also, the Clinical Global Impressions Severity Scale (CGI-S) was used for severity of psychotic symptoms. All the researchers were trained to use the scales and passed the conformance tests.

### Serum TNF-α and IL-1β measurements

Venous blood was collected between 7 and 8 a.m. following an overnight fast. All the blood samples were detected within 1 year after they were collected. The serum was separated and stored at −80°C until assayed. Serum TNF-α and IL-1β levels were examined by enzyme-linked immunosorbent assay (ELISA) using an available kit (Bender Med Systems GmbH Campus Vienna Biocenter 2A-1030 Vienna, Austria, Europe).

The standard and sample testing were performed using duplicate assays by the same investigator who knowed nothing about the study. The sensitivities for TNF-α and IL-1β were 0.3 and 5.0 pg/ml, respectively. The inter-assay coefficients were 8.6 and 8.1%, respectively. The intra-assay variation coefficients were 5.1 and 7.7%, respectively.

### Statistical analysis

The Kolmogorov–Smirnov tests were performed to test the normal distribution of data. Continuous variables were presented as mean ± standard deviation ($\overset{-}{\text{x}}$ ± SD). Categorical variables were recorded using frequencies and percentages. We used the one-way ANOVA for continuous variables and the χ^2^-test for categorical variables to test the between-group comparisons.

Since IL-1β was not normally distributed in the three groups, natural logarithmic transformation was performed for IL-1β. We used a univariate analysis of covariance (ANCOVA) controlling for age and gender to analyze TNF-α and IL-1β levels in the three groups. We used Bonferroni test to make *Post-hoc* comparisons between groups. Psychopathology on the PANSS, SANS, and CGI-S were compared between the two patient groups by one-way ANOVA and correlated with cytokine levels by calculating the partial correlation coefficients controlling for age, gender and course of illness. Differences at *p* < 0.05 level were considered to be significant. Statistical analysis were performed using SPSS 20.0 (SPSS Inc., Chicago, IL).

## Results

### Demographic data

Table 1 shows significant differences in age (*p* < 0.001), and education (*p* < 0.01) among three groups. There was also a statistically significant difference in course of illness (*p* < 0.001) between FEDN patients and CP. Gender among the three groups showed no difference (*p* > 0.05).

**Table 1:** Demographics of FEDN, chronic patients with schizophrenia and healthy controls.

	**FEDN(*n* = 69)**	**Chronic patients (*n* = 87)**	**Healthy controls (*n* = 61)**	**χ^2^ orF**	**df**	***P*-value**
Sex(M/F)	46/23	44/43	31/30	0.087	2	0.087
Age(years)	25.8 ± 5.9	32.9 ± 7.1	29.5 ± 6.7	22.0	2	< 0.001^**^
Education(years)	10.5 ± 2.9	11.0 ± 3.1	12.1 ± 2.6	5.2	2	0.006^**^
Course of disease(years)	0.8 ± 0.2	6.8 ± 1.4		55.0	154	< 0.001^**^
PANSS total score	84.9 ± 11.2	78.5 ± 7.1		18.9	154	< 0.001^**^
P subscore	18.2 ± 4.3	14.9 ± 2.6		37.4	154	< 0.001^**^
N subscore	25.6 ± 3.8	26.2 ± 3.0		5.0	154	0.241
G subscore	41.7 ± 7.6	37.4 ± 3.8		31.1	154	< 0.001^**^
SANS score	58.7 ± 13.5	64.7 ± 9.8		9.7	154	< 0.001^**^
CGI	5.5 ± 1.2	4.3 ± 0.8		15.9	154	< 0.001^**^
IL-1β(pg/ml)	1.7 ± 0.2	19.3 ± 11.3	8.3 ± 7.5	91.6^a^	2	< 0.001^**^^a^
TNF-α(pg/ml)	8.2 ± 2.0	28.1 ± 13.3	15.4 ± 7.0	90.5	2	< 0.001^**^

*FEDN, first-episode drug-naïve; PANSS, Positive and Negative Syndrome Scale; P subscore, positive subscore; N subscore, negative subscore; G subscore, general psychopathology subscore; SANS, Scale for the Assessment of Negative Symptoms; CGI, Clinical Global Impression Scale; IL-1β, Interleukin-1β; TNF-α, tumor necrosis factor-α*.

a *Refer to the results after natural logarithmic transformation was performed for IL-1β. Age of onset, education, IL-1 β and TNF-α among the three groups were compared by ANOVA*.

** *P < 0.001*.

### TNF-α, IL-1β and symptoms

All the blood samples were assayed within 1 year. There was no significant correlation between IL-1β or TNF-α levels and storage days (all *p* > 0.05). The PANSS total score, positive (P) and general psychopathology (G) subscores as well as CGI score showed significant differences between two patient groups (all *p* < 0.001) as exhibited in Table 1, with higher scores in FEDN patients than CP. However, the SANS score was significantly higher in chronic than FEDN patients (*p* < 0.001).

**Figure 1:**
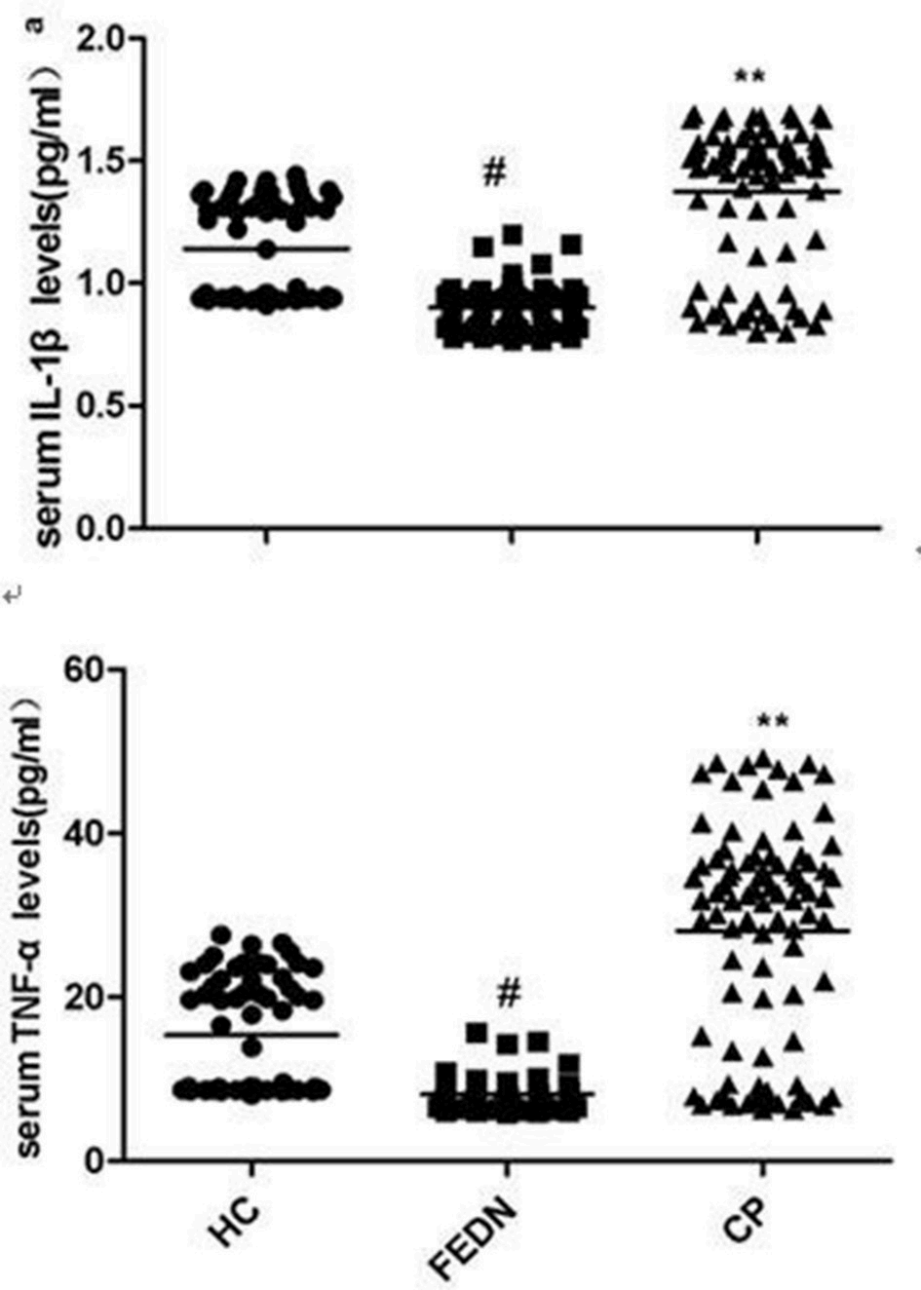
Serum TNF-α or IL-1β levels in FEDN (*n* = 69), CP (*n* = 87) and HC (*n* = 61).

As shown in Table 1 ANCOVA analysis revealed a significant difference in IL-1β (*F* = 87.5, *df* = 2, *p* < 0.0001)and TNF-α (*F* = 90.5, *df* = 2, *p* < 0.0001) levels among the three groups. *Post-hoc* analysis showed that TNF-α (*p* < 0.0001, Figure 1) and IL-1β (*p* < 0.0001, Figure 1) were significantly decreased in FEDN patients than both CP and healthy controls. Further, TNF-α (*p* < 0.0001, Figure 1) and IL-1β (*p* < 0.0001, Figure 1) were significantly higher in CP than in both FEDN patients and healthy controls.

### Correlation among TNF-α, IL-1β and symptoms

**Figure 2:**
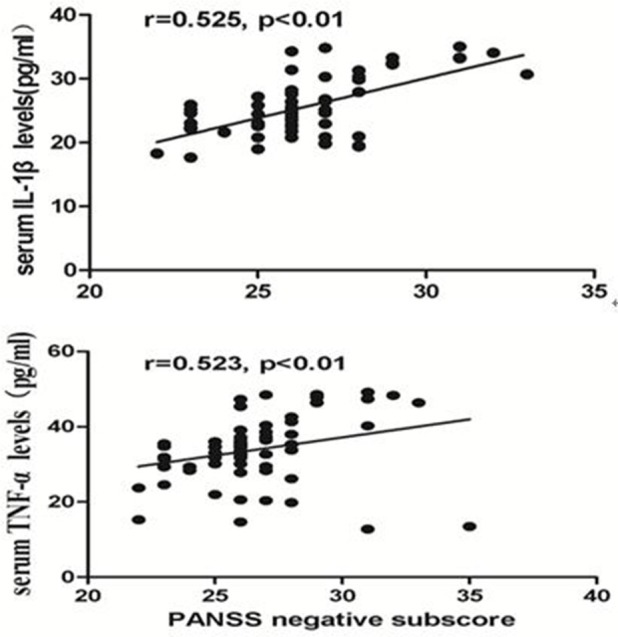
Moderate Significant positive correlation between the serum TNF-α or IL-1β levels and the PANSS negative subscore in chronic patients.

By using partial correlation analysis, we found a moderately positive correlation between IL-1β and the PANSS negative subscore (*r* = 0.525, *p* < 0.01, Figure 2), or between TNF-α and the PANSS negative subscore in CP (*r* = 0.523, *p* < 0.01, Figure 2), but no significant correlation between cytokine serum levels and PANSS positive subscore or general subscore (*p* > 0.05) was found in these patients. But we found no correlation between IL-1β or TNF-α and any clinical symptoms in FEDN patients (all *p* > 0.05). In addition, a significantly positive correlation between TNF-α and IL-1β was found in CP (*r* = 0.964, *p* < 0.001) but not in FEDN patients (*p* > 0.05).

## Discussion

This study had two important findings. Firstly, TNF-α and IL-1β serum levels were significantly higher in CP than both healthy controls and FEDN patients, while TNF-α and IL-1β serum levels were significantly decreased in the FEDN patients than both healthy controls and CP. Secondly, both TNF-α and IL-1β showed moderately positive correlations with the PANSS negative subscore in the CP but not in FEDN patients with schizophrenia.

For reason of the disease state, illness duration, medication, the timing of the assays and so on, the reports on levels of TNF-α and IL-1β in schizophrenia have been inconsistent. But our results are in accord with previous reports that TNF-α and IL-1β were increased in chronic schizophrenia patients (Söderlund et al., 2009; Miller et al., 2011; Monji et al., 2013; Song et al., 2014; Zhu et al., 2015). The increased TNF-α and IL-1β in chronic patients maybe related with the long-term antipsychotic treatments. Whether antipsychotic treatment may affect serum cytokine levels has been controversial (Potvin et al., 2008; Miller et al., 2011; Tourjman et al., 2013). Some review papers point out that antipsychotic medications have anti-inflammatory effects in schizophrenia. But no studies have reported the direct relationship between TNF-α or IL-1β and antipsychotics. The increased TNF-α and IL-1β in chronic patients may be related with older age, smoking or higher body mass indices (BMI). Interestingly, a positive correlation between TNF-α and IL-1β was found in CP but not in FEDN patients. Few studies has explored the interaction between TNF-α and IL-1β in patients with schizophrenia. Only one previous study reported a significant positive correlation between levels of TNF-α and IL-1β in schizophrenia patients (Liu et al., 2010). This supports that pro-inflammatory cytokines don't work independently but affect on the neuroimmunological network by means of mutual interactions. How the interaction between TNF-α and IL-1β is participated in the pathogenesis of schizophrenia warrants the further investigation.

Further, compared with healthy controls, a significant decrease in TNF-α and IL-1β levels were found in FEDN patients. These changes may be related with age, illness duration (Fawzi et al., 2011), cigarette smoking, disease state (Miller et al., 2011), the heterogeneity of schizophrenia, antipsychotic treatment (Davey et al., 2012), and comorbid obesity (Song et al., 2014), etc. This finding was inconsistent with the previous studies (Song et al., 2009; Müller et al., 2015). Interestingly, our results are in accordance with a recent study finding significantly higher IL-3 levels in CP but significantly fewer IL-3 levels in FEDN patients (Fu et al., 2016). They speculated that the reduced IL-3 levels in FEDN patients might be associated with neuronal apoptosis and abnormal early development of the CNS. In some situations, pro-inflammatory effects may have relation to important side effects, such as their responses to stress (Hinze-Selch et al., 2000; Zhang et al., 2005) and weight gain (Drzyzga et al., 2006). Some studies have proved that heightened stress is immunosuppressive (Adamo, 2012), and thus, the decreased TNF-α and IL-1β in our current study may be caused by stress in first episode schizophrenia, since the experience of acute psychosis in schizophrenia patients is stressful itself. The underlying mechanisms for the decreased TNF-α and IL-1β levels in FEDN patients with schizophrenia should be further investigated. In addition, we also found that both TNF-α and IL-1β showed moderately positive associations with the PANSS negative subscore in the CP. A recent study found a significant correlation between IL-3 levels and the PANSS G subscore only in CP(Fu et al., 2016). Another study also found that a significant decrease in IL-10 levels was reported in the FEDN patients and serum IL-10 was inversely correlated with the PANSS cognitive factor subscores, as well as with the PANSS negative symptom (Xiu et al., 2014). Taken together, these results point out that different cytokines may be related with clinical symptoms of schizophrenia.

Microglia are the resident macrophage in the brain and they are also the primary reservoirs of pro-inflammatory cytokines in the CNS (Monji et al., 2009). It is highly likely that the activated microglia may produce cytokines which probably cause toxicity to neurons and decrease in neurogenesis, which may be participated in the pathogenesis of negative symptoms in schizophrenia (Monji et al., 2013). In our current study, both TNF-α and IL-1β showed moderately positive associations with the PANSS negative subscore in the CP. As discussed above, TNF-α and IL-1β were participated in the processes of neurogenesis or white matter abnormalities, suggesting that altered TNF-α and IL-1β may be associated with negative symptoms of schizophrenia. A previous study found that the VNTR polymorphism in the IL-1RN gene may predict the improvement of negative symptom in schizophrenic patients which are treated with antipsychotic drugs (Mata et al., 2006). In our previous study, we found significant and persistent increases in the number of activated microglial cells and cytokines in an immune-related animal model that imitated negative symptoms in schizophrenia (Zhu et al., 2014a,b). We also found that minocycline, an inhibitor of microglial activation had significant efficacy for negative symptoms of schizophrenia (Liu et al., 2014). Taken all together, these findings suggest that the increased TNF-α and IL-1β, which may be caused by the activated microglia are related with negative symptoms of schizophrenia and anti-inflammatory may have therapeutic effects on clinical symptoms, especially on negative symptoms in schizophrenia.

The study has some limitations. Firstly, the sample size is relatively smaller. Secondly, we just measured only two cytokines. Previous studies have demonstrated that many cytokines are involved in immune dysfunction in schizophrenia. Therefore, further investigation will be needed to evaluate the role of other cytokines in psychopathologic mechanisms of schizophrenia. Thirdly, we did not collect some important clinical information, such as smoking, BMI and other data, which may affect the TNF-α and IL-1β levels in schizophrenia patients. For example, the popularity of smoking is much greater in schizophrenia patients than in the healthy population. One study reported that cigarette smoke played the harmful effects on human health by reason of its suppressive effects on the immune system (Zhang et al., 2008). Moreover, a previous study showed that smokers had lower IL-2 and IL-6 levels than non-smokers in chronic schizophrenia patients (Zhang et al., 2008). Unluckily, we did not gather smoking data in our current study, which should be added in future investigation. The role of smoking in altered cytokine levels in schizophrenia warrants further investigation.

## Conclusion

In summary, our data showed that TNF-α and IL-1β levels were decreased in FEDN patients, but elevated in CP. The increase of TNF-α and IL-1β levels may be related with the psychotropic drugs as well as the progression of the disease. The increased TNF-α and IL-1β were just moderately related with the negative symptoms in CP, but it is a helpful hint that there is a greater contribution of immune abnormality to the progression in this subgroup of patients and that immune modulating treatments may become a new strategy of therapy for this subgroup of patients.

## Author contributions

JZ: designed the study; FZ: wrote the protocol and the first draft of the manuscript; LZ and FL: collected the original data; RW, WG, and JO: undertook the statistical analysis; XZ: revised the draft of the manuscript. All authors contributed to and have approved the final manuscript.

### Conflict of interest statement

The authors declare that the research was conducted in the absence of any commercial or financial relationships that could be construed as a potential conflict of interest.
